# Total Synthesis of a Marine Alkaloid—Rigidin E

**DOI:** 10.3390/md10061412

**Published:** 2012-06-20

**Authors:** Banpeng Cao, Haixin Ding, Ruchun Yang, Xiaoji Wang, Qiang Xiao

**Affiliations:** Jiangxi Key Laboratory of Organic Chemistry, Jiangxi Science and Technology Normal University, Nanchang 330013, China; Email: caobanpeng@126.com (B.C.); dinghaixin@yahoo.cn (H.D.); ouyangruchun@yahoo.cn (R.Y.)

**Keywords:** pyrrolo[2,3-*d*]pyrimidine, alkaloids, total synthesis, domino reaction, marine natural products

## Abstract

In the present paper, we report an efficient total synthesis of a marine alkaloid, rigidin E. The key tetrasubstituted 2-amino-3-carboxamidepyrrole intermediate was synthesized by cascade Michael addition/intramolecular cyclization between *N*-(2-(4-(benzyloxy)phenyl)-2-oxoethyl)methanesulfonamide and 3-(4-(benzyloxy)phenyl)-2-cyano-*N*-methylacrylamide. Subsequent carbonylation with triphosgene catalyzed by I_2_ and deprotection of benzyl groups afforded rigidin E in 21% overall yield. This strategy has the merits of metal-free reactions, low cost, mild reaction protocols, and easy access to diversity-oriented derivatives for potential structure-activity relationship investigation.

## 1. Introduction

**Figure 1:**
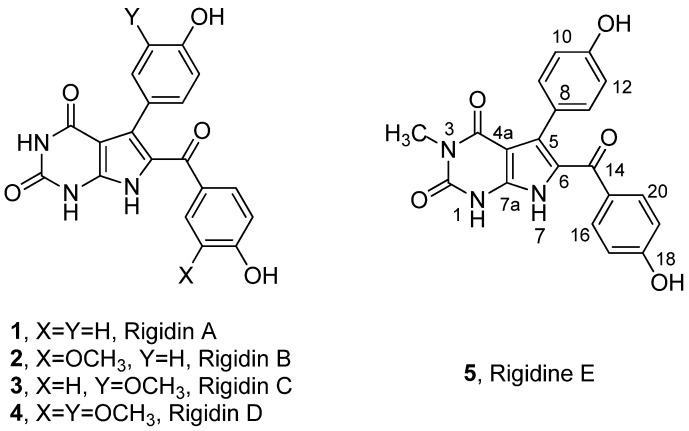
Structures of marine alkaloids rigidin A–E.

Rigidin A (**1**), a pyrrolo[2,3-*d*]pyrimidine alkaloid, was first isolated by Kobayashi *et al.* from Okinawa marine tunicate *Eudisromu* cf. *rigida* in 1990 [1]. Later on, a series of its analogues, Rigidin B–E (**2**–**5**) (Figure 1), which were consequently isolated from the same marine species [2], were found to have strong inhibitory activity against calmodulin brain phosphodiesterase [3]. The core structure of rigidin A–E is a tetrasubstituted pyrrole fused to a pyrimidine, which is an important structural subunit in a variety of biologically active compounds. In the past decades, much attention have been drawn on the pyrrolo[2,3-*d*]pyrimidine analogues for biological and pharmaceutical applications [4,5,6,7,8,9,10,11].

Due to the fact that the content of rigidins in localized tunicate species is very low (0.0015% wet weight), only very limited amount of rigidins could be isolated for biological study. Therefore, total synthesis of rigidin was employed to provide sufficient samples for their biological assays.

Currently, there are four published synthetic routes for the total synthesis of rigidins. In 1993, Edstrom *et al.* presented the first report of total synthesis of rigidin A (**1**) with 1,3-dibenzyl protected 6-chlorouracil in 26% overall yield [12]. After a S_N_2 substitution of the 6-chloro group with *N*-benzylglycine, the pyrrolo[2,3-*d*]pyrimidine skeleton was formed by reflux in acetic anhydride. The two substitutes at 5- and 6-position were then attached on Stille cross-coupling and Friedel-Crafts acylation respectively.

Soon after, Sakamoto and his co-workers reported the second strategy for total synthesis of rigidin A (**1**) in 1994 [13,14]. With a multi-substituted bromopyrimidine as starting material, the pyrrolo[2,3-*d*]pyrimidine core was built by Stille cross-coupling reaction with vinylstannane and subsequent acidic hydrolysis. The two substitutes were then introduced by similar reactions reported by Edstrom [12]. Rigidin A (**1**) was obtained in less than 10% overall yield.

In 2006, Gupton *et al.* reported the third total synthesis strategy for rigidin A (**1**) and rigidin E (**5**), which used a symmetrical vinamidinium salt to construct 2,4-disubstituted pyrrole [15]. After C-6 substitute was introduced by Friedel-Crafts acylation, the pyrimidine moiety was constructed to accomplish the total synthesis of rigidin A (**1**) and rigidin E (**5**).

The first and second synthetic routes employed substituted pyrimidine as the starting material and subsequently constructed the pyrrole moiety. In contrast, the third route constructed multi-substituted pyrrole moiety before pyrimidine formation. All three routes suffered from harsh reaction conditions, expensive Palladium-based catalyst, lengthy route and lack of variability for diversity-oriented derivatives.

In 2011, Magedov reported that tetra- and pentasubstituted 2-aminopyrroles can be prepared via multi-component reactions of structurally diverse aldehydes and *N*-(aryl-, heteroaryl-, alkylsulfonamido)acetophenones with cyanoacetic acid derivatives, such as malononitrile and cyanoacetate [16]. Furthermore, this methodology was used successfully in total synthesis of rigidins A–D in moderate overall yield, which is the fourth total synthetic route [16]. So far, this is the most efficient total synthetic strategy. However, this protocol’s generality and reproducibility still needs to be proved. We describe herein an improved and practical synthetic route for the total synthesis of rigidin E.

## 2. Results and Discussion

Rigidin E contains many *H*-bond donor/acceptors, which may coordinate with heavy metal ions and form metal-rigidin complexes. The resulting metal contamination cannot usually be easily removed by a normal purification process. This promoted us to develop a metal-free strategy. 

**Figure 2:**
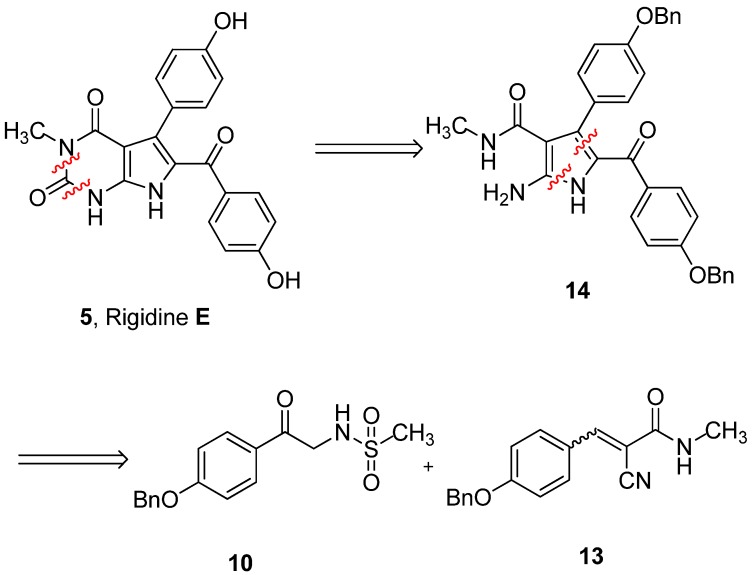
Retrosynthetic analysis of rigidin E.

Our retrosynthetic analysis is showed in Figure 2. Principally, rigidin E may be synthesized from rigidin A through regioselective methylation at N-3. This reaction is very difficult to realize due to the three NH groups in rigidin E. Therefore, it is preferable to introduce the methyl group before constructing the tetrasubstituted 2-aminopyrrole.

**Scheme 1:**
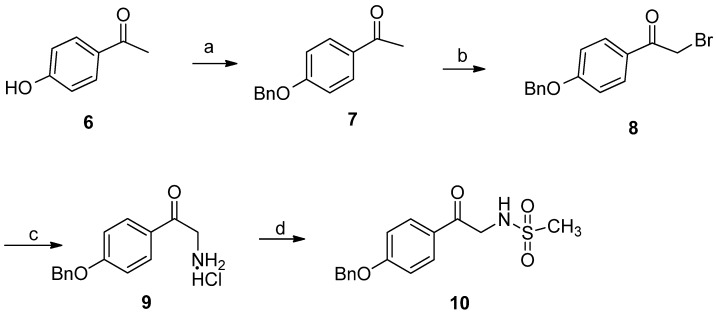
Synthesis of methanesulfonamide **10**. Reagents and conditions: (**a**) K_2_CO_3_, acetone, BnBr, 0 °C, 98%; (**b**) phenyltriethylammonium tribromide, THF, 80%; (**c**) hexamethylenetetramine, chlorobenzene, then conc. HCl; (**d**) MsCl, Et_3_N, acetone/H_2_O 2:1, 60% over two steps.

Regioselective bromination of acetophenone **7** with phenyltrimethylammonium tribromide in anhydrous THF afforded 2-bromoacetophenone **8** in 80% yield [17]. 2-Amino-acetophenone **9 **was synthesized using hexamethylenetetramine as an NH_2_ source in anhydrous chlorobenzene. Subsequent reaction with methanesulfonyl chloride gave methanesulfonamide **10** in 60% yield over two steps (Scheme 1).

**Scheme 2:**
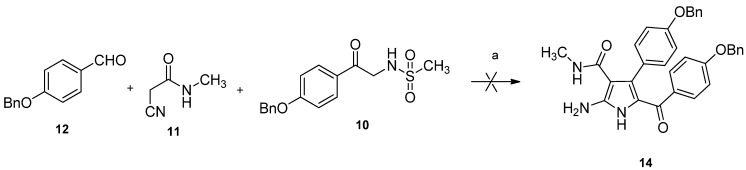
Three-components reaction for synthesis the key intermediate **14** failed. Reagents and conditions: (**a**) K_2_CO_3_, EtOH, reflux.

In order to introduce the methyl group to N-3, 2-cyano-*N*-methylacetamide **11** was synthesized from ethyl cyanoacetate and methylamine in 90% yield [18,19]. Attempts to synthesize key intermediate **14** using the reported three-components reaction of compound **10**, **11**, and **12** proceeded in very low yield and was complicated by undesired byproducts (Scheme 2). As reported by Magedov [16], Knoevenagel adduct **13** may be the intermediate in the three-component reaction. We speculated that the intermediate **13** could not form efficiently because of the higher p*K*_a_ of the methylene of *N*-methylacetamide compare to *N*-unsubstituted acetamide. Therefore, it is possible to solve this problem through synthesis of the intermediate **13 ** independently.

**Scheme 3:**
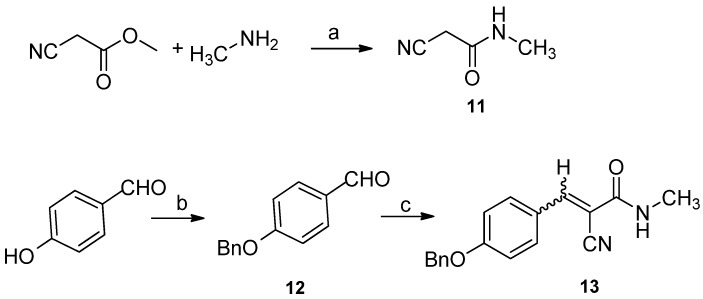
Synthesis of *N*-methylacrylamide **13**. Reagents and conditions: (**a**) methylamine, H_2_O, 90%; (**b**) K_2_CO_3_, acetone, BnBr, 0 °C, 95%; (**c**) **11**, piperidine, toluene, 80%.

After Knoevenagel condensation of 2-cyano-*N*-methylacetamide **11** and 4-(benzyloxy)benzaldehyde **12** using piperidine as a catalyst, 3-(4-(benzyloxy)phenyl)-2-cyano-*N*-methylacrylamide **13** was obtained in 80% yield as a mixture of *E/Z* in about 1:1 ratio (Scheme 3) [20]. Then, a variation of the reported three-components reaction was conducted to synthesize the precursor **14**.

After a careful screening of reaction conditions, the key intermediate **14** was successfully prepared by a cascade Michael addition/intermolecular cyclization between *N*-methylacrylamide **13** and methanesulfonamide **10** in one pot, with K_2_CO_3_ as base and ethanol as solvent under refluxing conditions. The present result proves that phenyl-2-cyanoacrylamide like Knoevenagel adduct was the intermediate for cyclization in the reported three-component reaction [21].

**Scheme 4:**
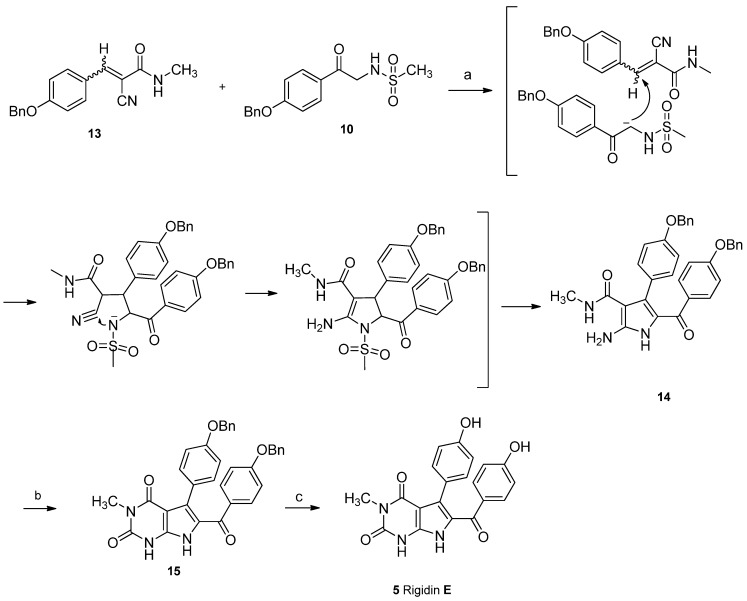
Total synthesis of rigidin E. Reagents and conditions: (**a**) K_2_CO_3_, EtOH, reflux, 50%; (**b**) triphosgene, I_2_, THF, 60%; (**c**) TMSCl, NaI, CH_3_CN, 88%.

Carbonylation with oxalyl chloride in diglyme failed to give pyrimidinedione **15** [16]. Various reagents and conditions were tested, and triphosgene was found ideal to promote the I_2_-catalyzed cyclization in anhydrous THF to give pyrimidinedione **15** in 60% yield [22,23]. The final deprotection of benzyl groups using catalytic hydrogenation resulted in a complicated mixture. The reason might be the accompanied reduction of carbonyl at C-14. Finally, the protective groups were successfully removed by TMSI *in situ* prepared by TMSCl and NaI to afford rigidin E (**5**) in 88% yield (Scheme 4). All spectral data are consistent with those of the reported natural product [3].

## 3. Experimental Section

### 3.1. General

All reagents and catalysts were purchased from commercial sources (Acros or Sigma Aldrich) and used without purification. MeCN, chlorobenzene and DCM were dried with CaH_2 _and distilled prior to use. THF was dried with LiAlH_4_ and distilled prior to use. Thin layer chromatography was performed using silica gel GF-254 plates (Qing-Dao Chemical Company, China) with detection by UV (254 nm) or charting with 10% sulfuric acid in ethanol. Column chromatography was performed on silica gel (200–300 mesh, Qing-Dao Chemical Company, China). NMR spectra were recorded on a Bruker AV400 spectrometer, and chemical shifts (δ) are reported in ppm. ^1^H NMR and ^13^C NMR spectra were calibrated with TMS as internal standard, and coupling constants (*J*) are reported in Hz. The ESI-HRMS were obtained on a Bruker Dalton microTOFQ II spectrometer in positive ion mode. Melting points were measured on an electrothermal apparatus uncorrected.

### 3.2. 1-(4-(Benzyloxy)phenyl)-2-bromoethanone **8**

To a solution of 1-(4-(benzyloxy)phenyl)ethanone **7** (2.26 g, 10 mmol) in anhydrous THF (20 mL) was added phenyltrimethylammonium tribromide (3.76 g, 10 mmol). The resulting solution was stirred at room temperature. A white precipitate was formed and the solution became yellowish over several minutes. After 30 min, the mixture was poured into ice water (50 mL) and extracted with ethyl acetate (20 mL × 3). The combined organic layer was dried over anhydrous Na_2_SO_4_, filtered, and concentrated. The crude product was recrystallized from ethanol (15 mL) to afford the title product **8** (2.43 g, 80% yield).

MP: 67–69 °C; *R_f_* = 0.30 (PE–EtOAc, 50:1). ^1^H NMR (400 MHz, CDCl_3_): δ = 4.40 (s, 2H, CH_2_Br), 5.14 (s, 2H, C_6_H_5_CH_2_), 7.03 (d, *J* = 8.8 Hz, 2H, C_6_H_4_, H-3,5), 7.35–7.44 (m, 5H, C_6_H_5_), 7.96 (d, *J* = 8.8 Hz, 2H, C_6_H_4_, H-2,4). ^13^C NMR (100 MHz, CDCl_3_): δ = 30.7, 70.2, 114.9, 127.5, 128.3, 128.7, 130.6, 131.4, 135.9, 163.3, 190.0. MS (ESI): *m/z* = 304.8 [M + H]^+^.

### 3.3. *N*-(2-(4-(Benzyloxy)phenyl)-2-oxoethyl)methanesulfonamide **10**

1-(4-(Benzyloxy)phenyl)-2-bromoethanone **8** (3.04 g, 10 mmol) was dissolved in anhydrous chlorobenzene (8.5 mL). The solution was added dropwise to a solution of hexamethylenetetramine (1.15 g, 11 mmol) in anhydrous chlorobenzene (10 mL) at 30 °C. The reaction mixture was stirred at 30 °C for 4 h and filtered. The filter cake was washed with ethanol (10 mL) and dried *in vacuo*. The filter cake was redissolved in a mixture of concentrated hydrochloric acid (10 mL) and ethanol (20 mL) with vigorous stirring. The resulting reaction mixture was stirred occasionally under N_2_ atmosphere for 48 h. The reaction mixture was cooled to 0 °C and filtered to give compound **9**. It was redissolved in H_2_O/acetone (1:2, 600 mL). Then methanesulfonyl chloride (1.71 g, 15 mmol) was added to the mixture. The mixture was placed in an ice bath and triethylamine (2.53 g, 25 mmol) was added dropwise over 30 min. Then acetone (14 mL) was added to the above mixture. The reaction mixture was stirred at room temperature for 10 h, and then the volatiles were evaporated under reduced pressure. The solid started to precipitate. To the obtained slurry were added ethyl acetate (20 mL) and saturated NH_4_Cl (20 mL) aqueous solution. The organic layer was separated and washed with saturated NaHCO_3_ (20 mL) aqueous solution and brine (20 mL), dried with anhydrous MgSO_4_, and concentrated to afford the title product **10** (1.91 g, 60% yield).

Mp: 138–140 °C; *R_f_* = 0.43 (CH_3_OH–CH_2_Cl_2_, 1:50). ^1^H NMR (400 MHz, CDCl_3_): δ = 2.99 (s, 3H, CH_3_), 4.61 (s, 2H, CH_2_NH), 5.15 (s, 2H, C_6_H_5_CH_2_), 5.32–5.49 (b, 1H, NH), 7.04 (d, *J* = 8.8 Hz, 2H, C_6_H_4_, H-3,5), 7.25–7.42 (m, 5H, C_6_H_5_), 7.91 (d, *J* = 8.8 Hz, 2H, C_6_H_4_, H-2,4). ^13^C NMR (100 MHz, CDCl_3_): δ = 40.7, 48.8, 70.3, 115.1, 126.9, 127.5, 128.4, 128.7, 130.3, 135.8, 163.7, 191.6. MS (ESI): *m/z* = 342.5 [M + Na]^+^.

### 3.4. 4-(Benzyloxy)benzaldehyde **12**

To a mixture of 4-hydroxybenzaldehyde (1.22 g, 10 mmol) and K_2_CO_3_ (1.45 g, 10.5 mmol) in acetone (10 mL) was added benzyl bromide (1.80 g, 10.5 mmol). The resulting mixture was heated to reflux for 3 h. After cooling, the reaction mixture was poured into water (5 mL) and extracted with Et_2_O (5 mL × 3). The combined organic layers were washed with brine (20 mL), dried over anhydrous MgSO_4_, filtered, and concentrated under reduced pressure to give a white solid. The solid was washed with 95% ethanol (2 mL) to afford pure product **12** (2.01 g, 95% yield). 

MP: 69–71 °C; *R_f_* = 0.49 (PE–EtOAc, 4:1). ^1^H NMR (400 MHz, CDCl_3_): δ = 5.15 (s, 2H, C_6_H_5_CH_2_), 7.08 (d, *J* = 8.4 Hz, 2H, C_6_H_4_, H-3,5), 7.35–7.45 (m, 5H, C_6_H_5_CH_2_), 7.84 (d, *J* = 8.4 Hz, 2H, C_6_H_4_, H-2,4), 9.89 (s, 1H, CHO). ^13^C NMR (100 MHz, CDCl_3_): δ = 70.6, 115.5, 127.8, 128.6, 129.1, 130.5, 132.3, 136.3, 164.1, 191.1. MS (ESI): *m/z* = 213.2 [M + Na]^+^.

### 3.5. 2-Cyano-*N*-methylacetamide

To an ice cooled solution of methylamine (2.17 g, 75 mmol) in water, ethyl cyanoacetate (5.65 g, 50 mmol) was added dropwise. The solution was stirred at room temperature for 3 h. Most of the solvent were evaporated under reduced pressure. The obtained solid was filtered and redissolved in ethyl acetate (200 mL). The solution was dried over anhydrous Na_2_SO_4_. The solvent was evaporated to give the title product (4.14 g, 90% yield). 

MP: 81–83 °C. ^1^H NMR (400 MHz, DMSO): δ = 2.58 (d, *J* = 4.4 Hz, 3H, CH_3_), 3.57 (s, 2H, CH_2_), 8.14 (s, 1H, NH). ^13^C NMR (100 MHz, DMSO-*d*_6_): δ = 24.6, 25.4, 115.6, 161.9. MS (ESI): *m/z* = 99.4 [M + H]^+^.

### 3.6. 3-(4-(Benzyloxy)phenyl)-2-cyano-*N*-methylacrylamide **13**

To a mixture of 4-(benzyloxy)benzaldehyde **12** (2.12 g, 10 mmol) and piperidine (34 mg, 0.4 mmol) in toluene (10 mL) was added 2-cyano-*N*-methylacetamide (1.08 g, 11 mmol). The resulting mixture was heated to reflux for 15 h. After cooling, the reaction mixture was filtered. The filter cake was washed with 95% ethanol (20 mL), and then dried *in vacuo* to afford the title product **13** (2.34 g, 80% yield).

MP: 170–172 °C; *R_f_* = 0.37 (PE–EtOAc, 2:1). ^1^H NMR (400 MHz, DMSO-*d*_6_): δ = 2.72 (d, *J* = 4.4 Hz, 3H, CH_3_), 5.19 (s, 2H, CH_2_), 7.18 (d, *J* = 8.8 Hz, 2H, C_6_H_4_, H-3,5), 7.31–7.46 (m, 5H, C_6_H_5_), 7.95 (d, *J* = 8.8 Hz, 2H, C_6_H_4_, H-2,4), 8.08 (s, 1H, NH), 8.27 (d, *J* = 4.4 Hz, 1H, CH_2_). ^13^C NMR (100 MHz, DMSO-*d*_6_): δ = 27.2, 70.0, 103.0, 116.0, 117.4, 125.1, 128.4, 128.5, 129.0, 132.9, 136.8, 150.4, 162.0. MS (ESI): *m/z* = 315.1 [M + H]^+^.

### 3.7. 2-Amino-5-(4-(benzyloxy)benzoyl)-4-(4-(benzyloxy)phenyl)-*N*-methyl-1h-pyrrole-3-carboxamide **14**

To a mixture of 3-(4-(benzyloxy)phenyl)-2-cyano-*N*-methylarcylamide **13** (3.21 g, 11 mmol) and K_2_CO_3_ (0.69 g, 5 mmol) in ethanol (10 mL) was added *N*-(2-(4-(benzyloxy)phenyl)-2-oxoethyl)methanesulfonamide **10** (3.19 g, 10 mmol). The resulting mixture was refluxed for 12 h under N_2_ atmosphere. After the solvent was evaporated, the residue was purified by column chromatography with CH_3_OH/CH_2_Cl_2_ = 1:60 to afford the title product **14** (2.66 g, 50% yield).

MP 247–249 °C; *R_f_* = 0.40 (CH_3_OH–CH_2_Cl_2_, 1:60). ^1^H NMR (400 MHz, DMSO-*d*_6_): δ = 2.46 (d, *J* = 4.8 Hz, 3H, CONHCH_3_), 5.01 (s, 2H, C_6_H_4_OCH_2_C_6_H_5_), 5.03 (s, 2H, CH_2_), 5.24 (b, 1H), 6.25 (s, 2H, NH_2_), 6.62 (d, *J* = 8.8 Hz, 2H), 6.77 (d, *J* = 8.4 Hz, 2H), 6.94 (d, *J* = 8.4 Hz, 2H), 7.09 (d, 2H, *J* = 8.8 Hz), 7.33–7.36 (b, 10 H), 10.80 (s, 1H). ^13^C NMR (100 MHz, DMSO-*d*_6_): δ = 25.7, 69.8, 99.2, 113.6, 114.7,121.5, 126.5, 127.8, 128.2, 128.7, 130.2, 131.0, 132.1, 132.2, 137.0, 137.2, 148.8, 158.2, 159.8, 166.3, 183.3. MS (ESI): *m/z* = 570.2 [M + K]^+^.

### 3.8. 6-(4-(Benzyloxy)benzoyl)-5-(4-(benzyloxy)phenyl)-3-methyl-1*H*-pyrrolo[2,3-*d*]pyrimidine-2,4(3*H*,7*H*)-dione **15**

A mixture of pyrrolocarboxamide **14** (5.31 g, 10.0 mmol), triphosgene (1.48 g, 5.0 mmol) and iodine (0.127 g, 0.5 mmol) in anhydrous THF (10 mL) was heated to reflux for 20 h before the volatiles were removed *in vacuo*. The residue was dissolved in ethyl acetate (10 mL) and washed with brine (10 mL), dried over anhydrous MgSO_4_, and purified by column chromatography (MeOH/CH_2_Cl_2_ = 1:10) to afford the title product **15** (3.34 g, 60% yield).

MP: 226–228 °C; *R_f_* = 0.35 (MeOH–CH_2_Cl_2_, 1:10). ^1^H NMR (400 MHz, DMSO-*d*_6_): δ = 3.14 (s, 3H, CH_3_), 5.00 (s, 2H, CH_2_), 5.03 (s, 2H), 6.67–6.73 (m, 4H), 7.03–7.05 (m, 2H), 7.28–7.35 (m, 12H), 11.65 (s, 1H), 11.96 (s, 1H). ^13^C NMR (100 MHz, DMSO-*d*_6_): δ = 27.2, 69.7, 69.9, 98.4, 113.7, 114.2, 125.1, 125.7, 127.9, 128.0, 128.2, 128.4, 128.9, 130.9, 131.7, 132.7, 136.9, 137.5, 140.1, 151.2, 158.0, 159.5, 161.4, 185.7. MS (ESI): *m/z* = 596.2 [M + Na]^+^.

### 3.9. Rigidin E **5**

A mixture of 6-(4-(benzyloxy)benzoyl)-5-(4-(benzyloxy)phenyl)-3-methyl-1*H*-pyrrolo[2,3-*d*]pyrimidine-2,4(3*H*,7*H*)-dione **15** (1.114 g, 2 mmol) and NaI (9.0 g, 60 mmol) in CH_3_CN (20 mL) under N_2_ was stirred at room temperature for 20 min. Chlorotrimethylsilane (17.8 g, 60 mmol) was then added. The mixture was stirred at 60 °C for 18 h before the volatiles were removed *in vacuo*. The residue was extracted with petroleum ether (10 mL × 3) in order to remove benzyl iodide, and then treated with water (5 mL) and ethyl acetate (10 mL). The mixture was stirred vigorously at room temperature for 2 h and extracted with more ethyl acetate (40 mL × 2). The combined organic layers were washed with brine (5 mL) containing a small amount of sodium thiosulfate, dried over anhydrous MgSO_4_, and purified by column chromatography (PE/Ethyl Acetate/MeOH/AcOH = 9:18:1:1) to afford the title product rigidin E (**5**) (664 mg, 88% yield).

MP > 300 °C; *R_f_* = 0.31 (PE–Ethyl Acetate–MeOH–AcOH = 9:18:1:1). ^1^H NMR (400 MHz, DMSO-*d*_6_): δ = 3.14 (s, 3H, CH_3_), 6.44 (t, *J* = 8.0 Hz, 4H), 6.93 (d, *J* = 7.6 Hz, 2H), 7.27 (d, *J* = 8.0 Hz, 2H). ^13^C NMR (100 MHz, DMSO-*d*_6_): δ = 27.2, 98.2, 114.2, 114.7, 123.4, 125.3, 128.7, 129.2, 132.0, 132.8, 152.4, 156.8, 159.9, 161.0, 185.7. HRMS–ESI: *m/z* [M + H]^+^ calcd for C_20_H_15_N_3_O_5_: 378.1012; found: 378.1098.

## 4. Conclusions

In conclusion, we have developed an efficient approach for the total synthesis of rigidin E using readily available starting materials and routine chemical transformations. This strategy is especially useful to access different N-3 substituted rigidin derivatives. 

## Supplementary Files

Supplementary File 1: PDF-Document (PDF, 689 KB)
