# Supplementary material for: Total Synthesis of a Marine Alkaloid—Rigidin E
Source: Mar Drugs. 2012 Jun 20;10(6):1412–21. doi: 10.3390/md10061412 (PMC3397449; doi:10.3390/md10061412)

Supplementary Information

Figure S1. ^{1}H NMR spectrum of compound 8

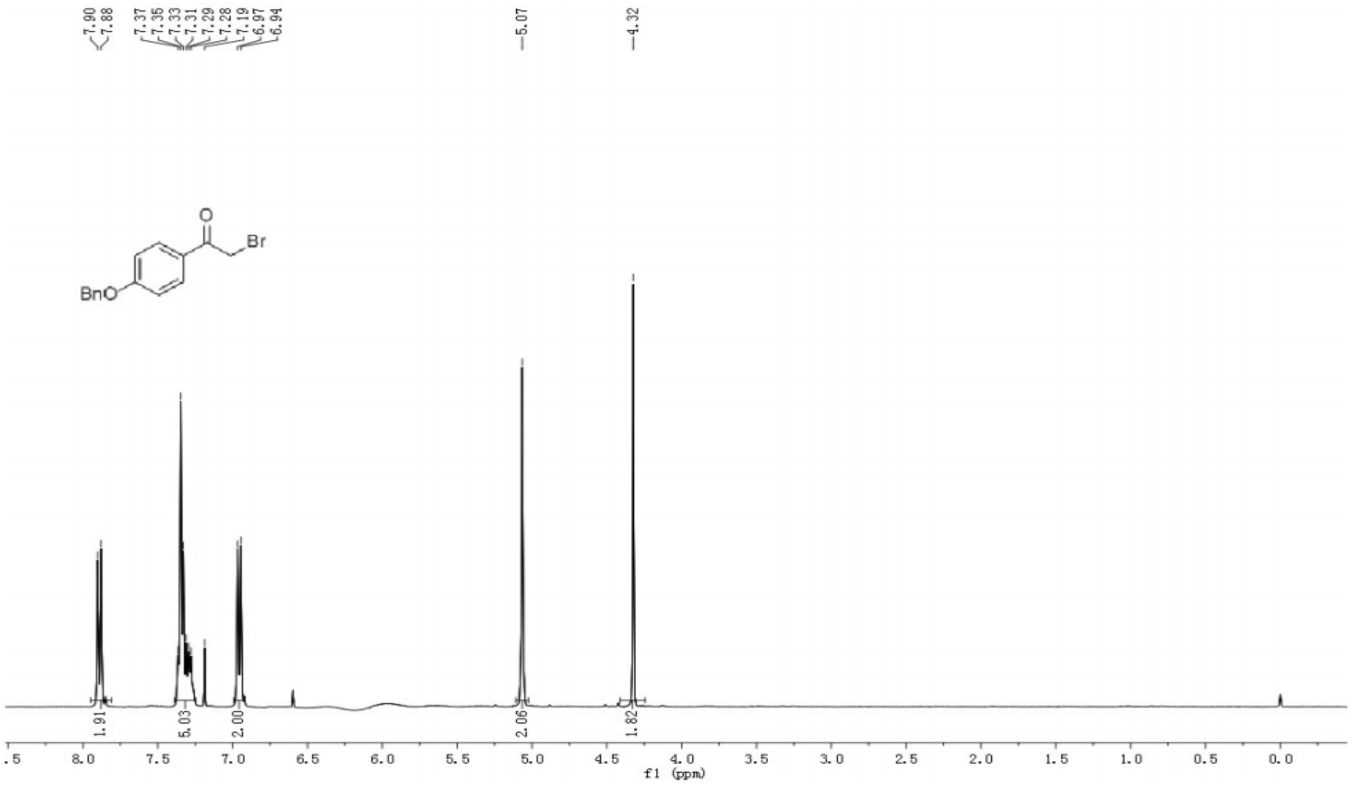

Figure S2. ^{13}C NMR spectrum of compound 8

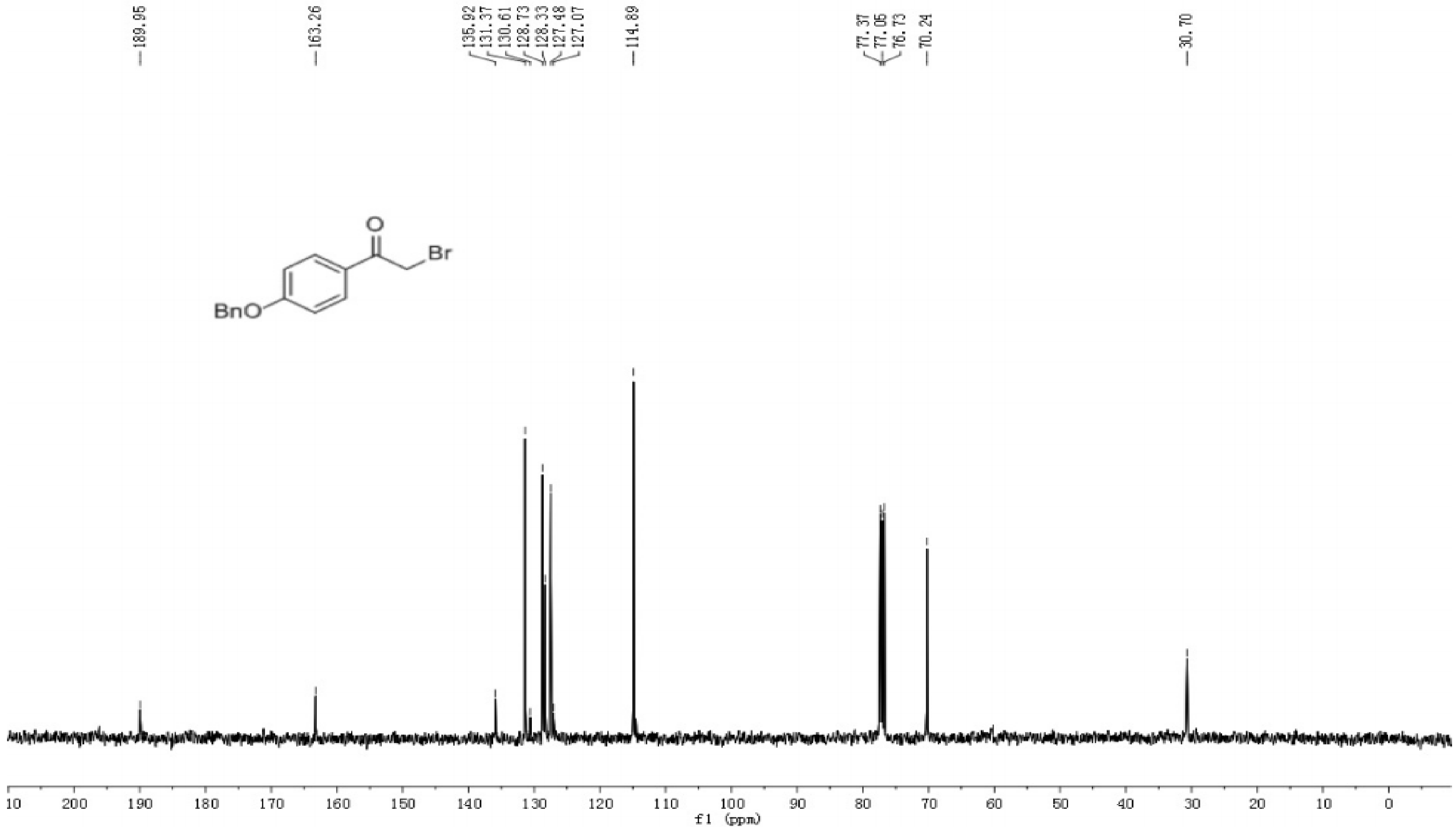

Figure S3. ^{1}H NMR spectrum of compound 10

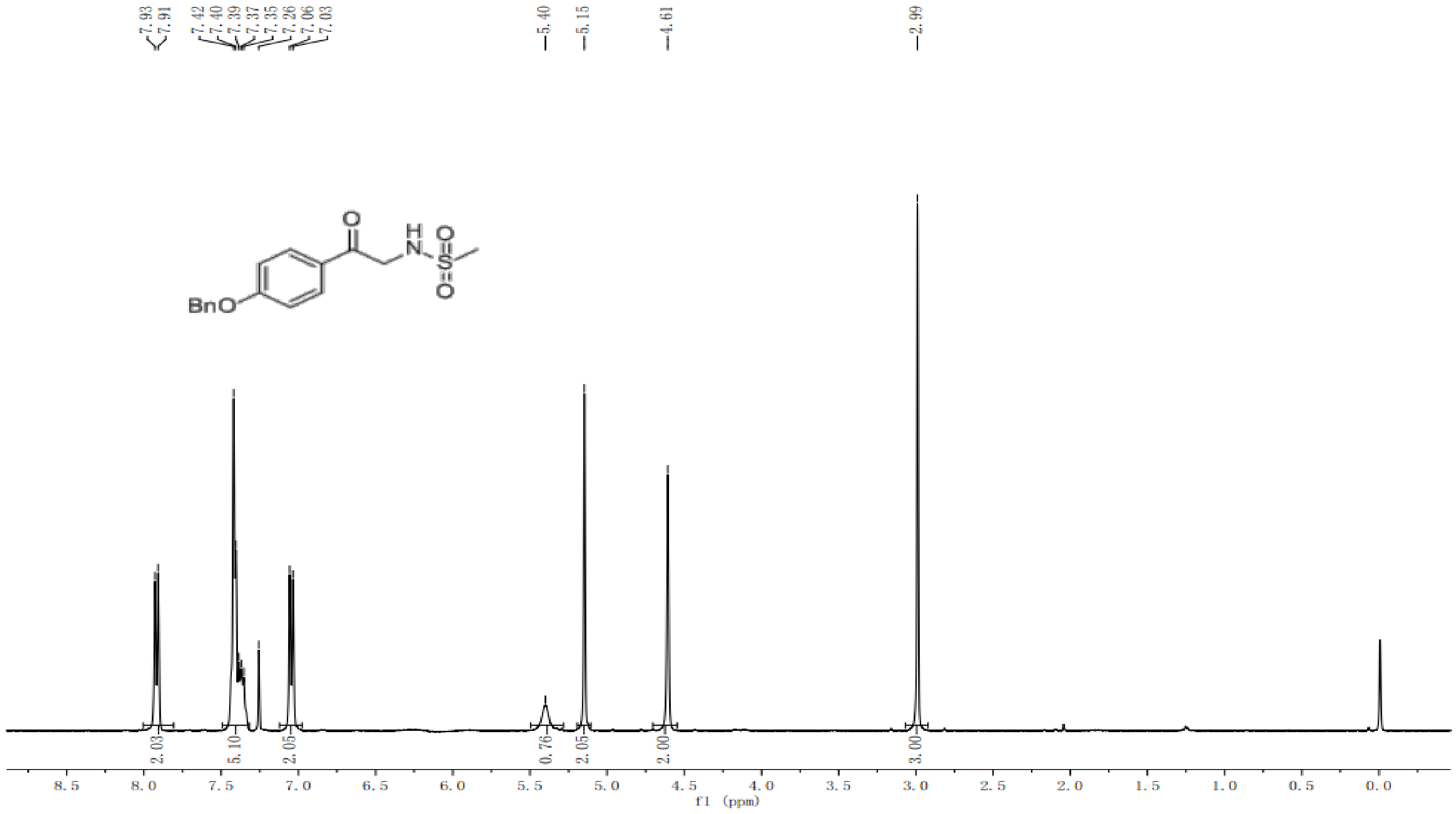

Figure S4. ^{13}C NMR spectrum of compound 10

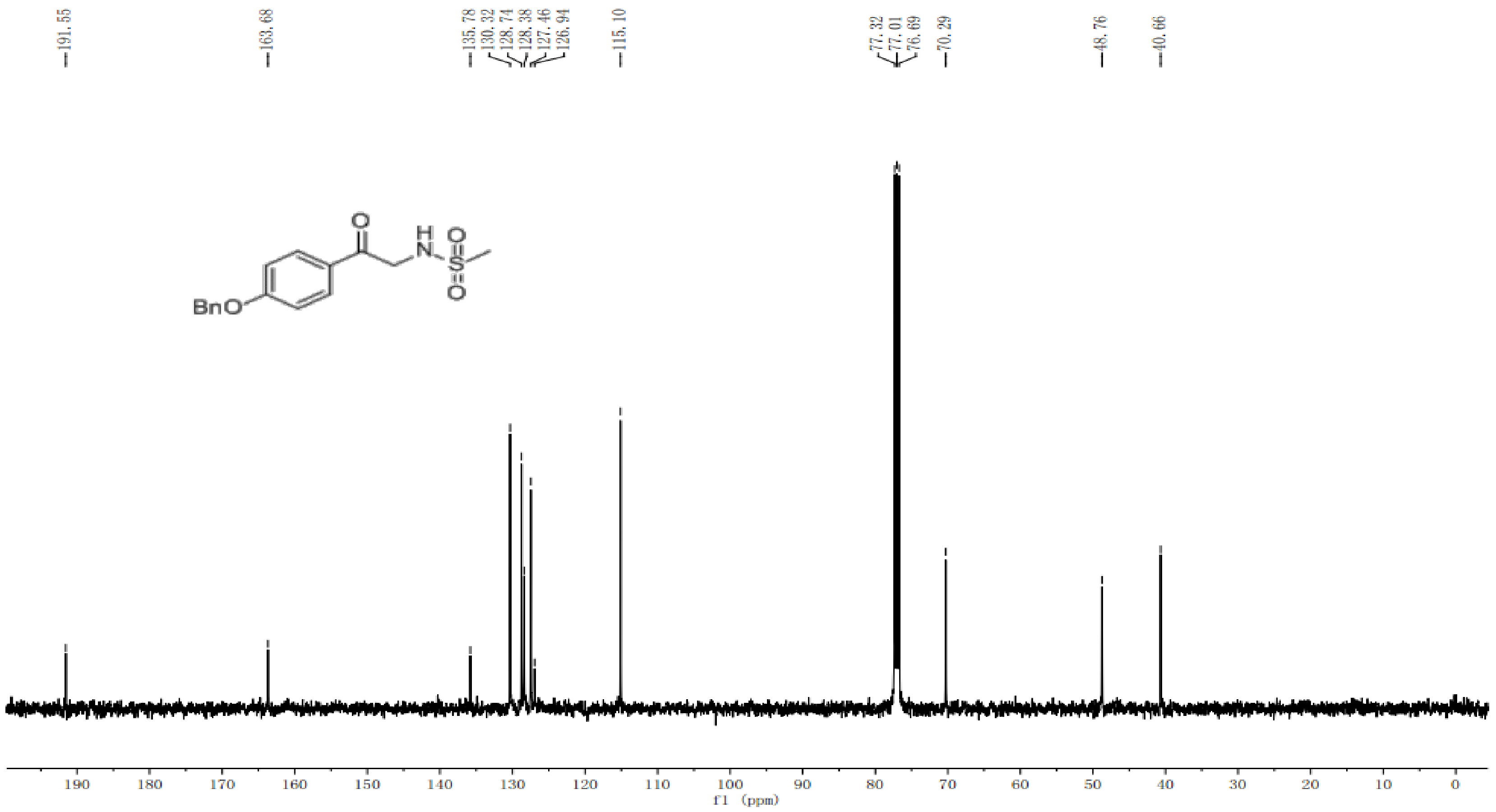

Figure S5. ¹H NMR spectrum of compound 12

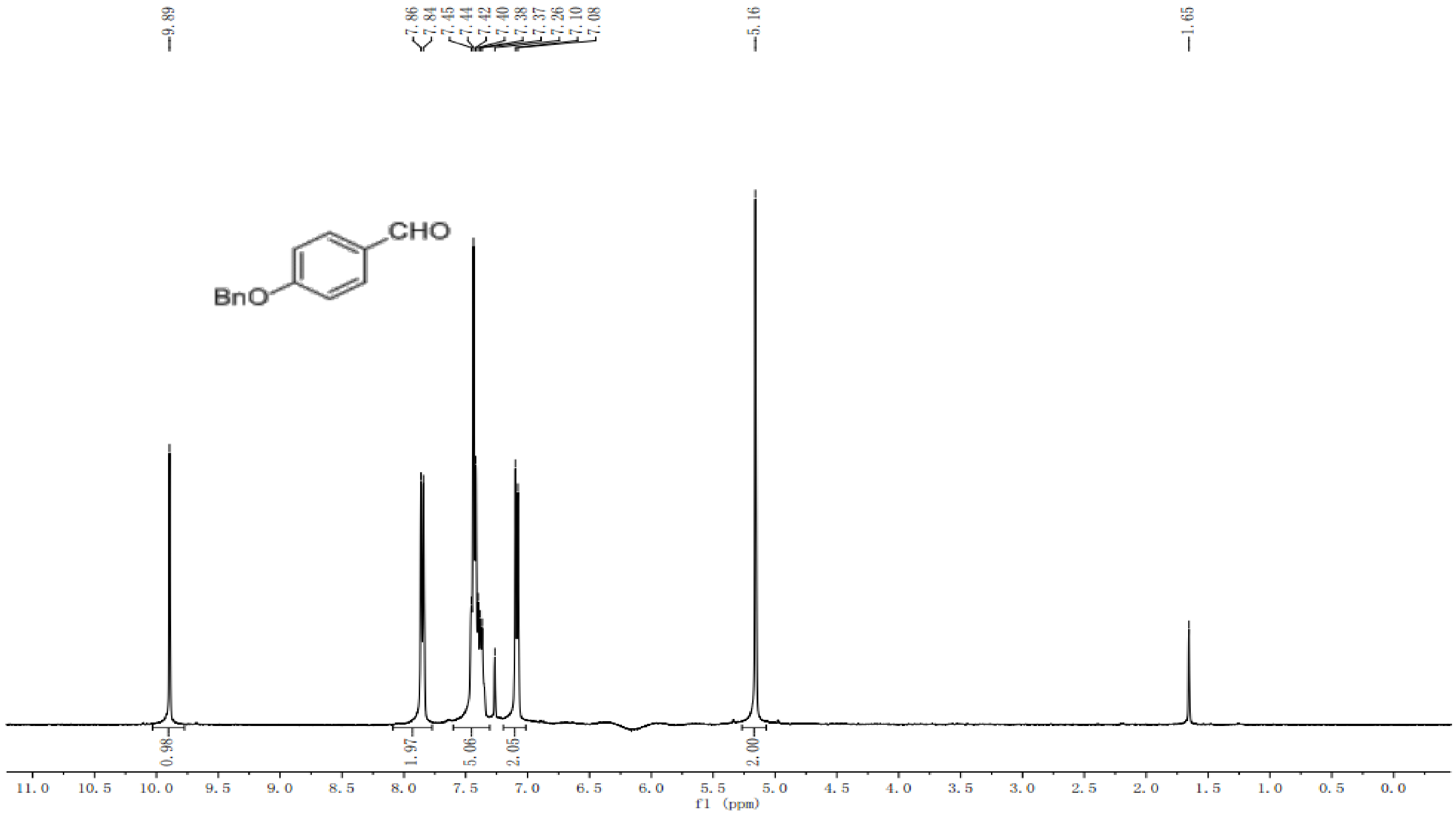

Figure S6. ^{13}C NMR spectrum of compound 12

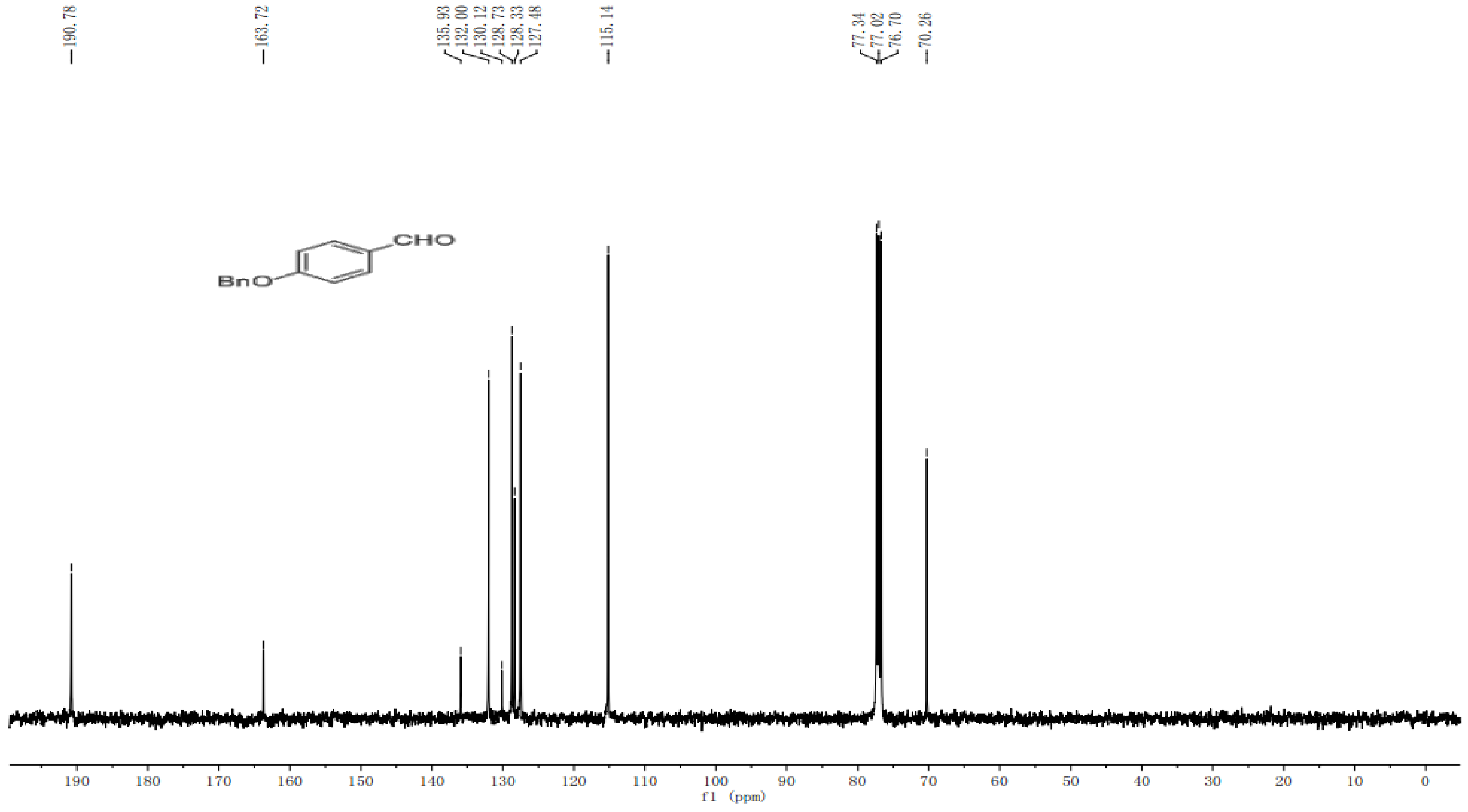

Figure S7. ^{1}H NMR spectrum of compound 11

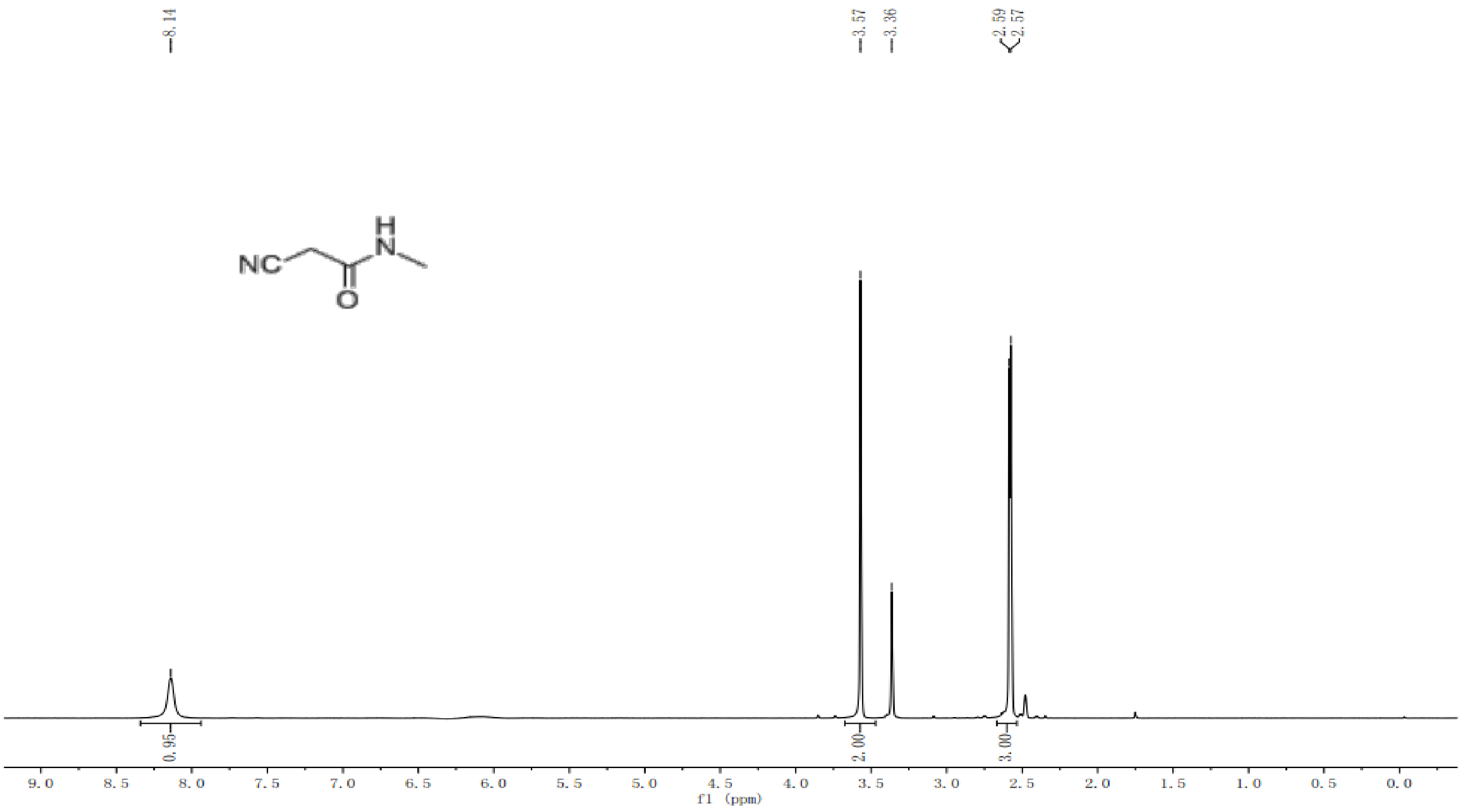

Figure S8. ^{13}C NMR spectrum of compound 11

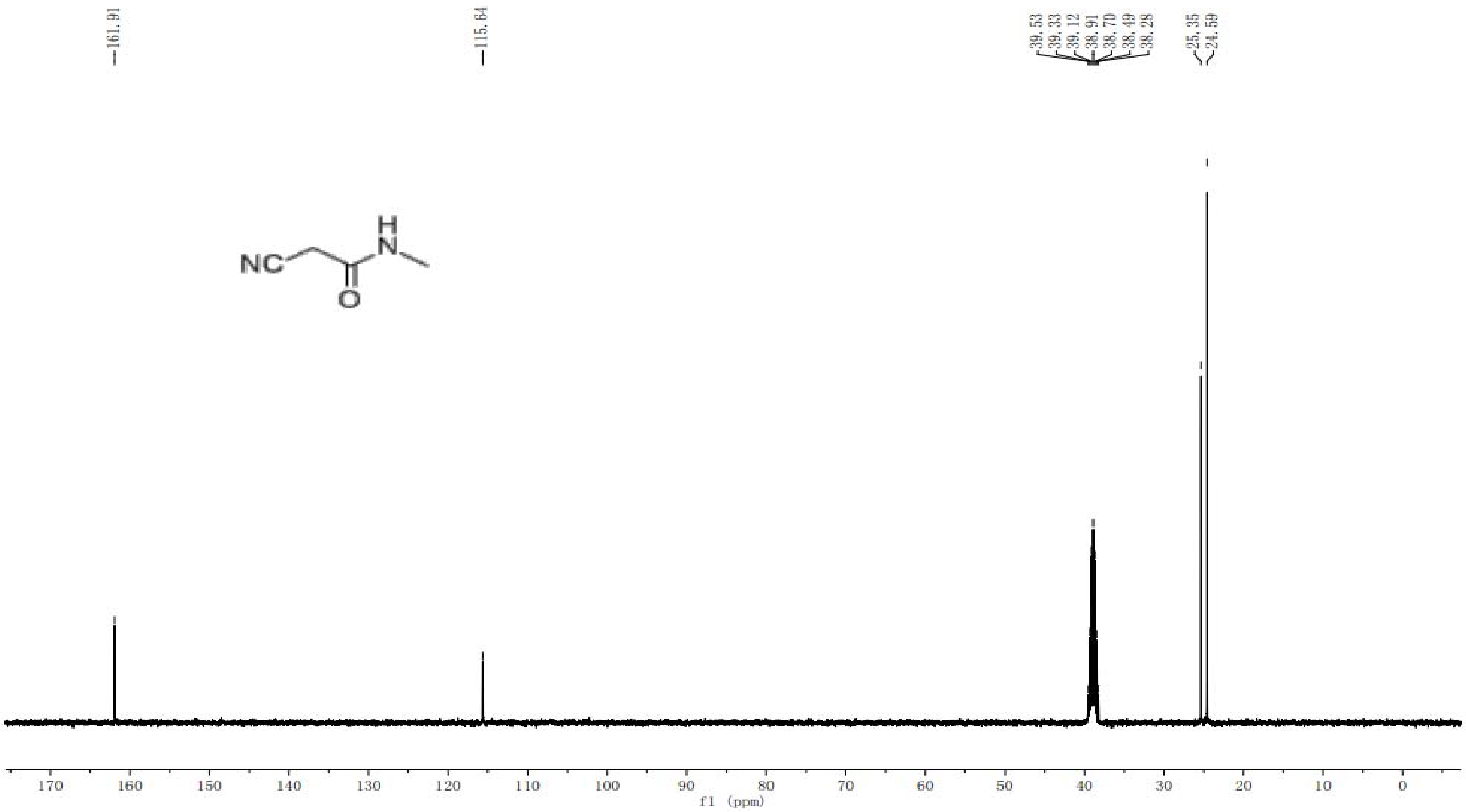

Figure S9. ^{1}H NMR spectrum of compound 13

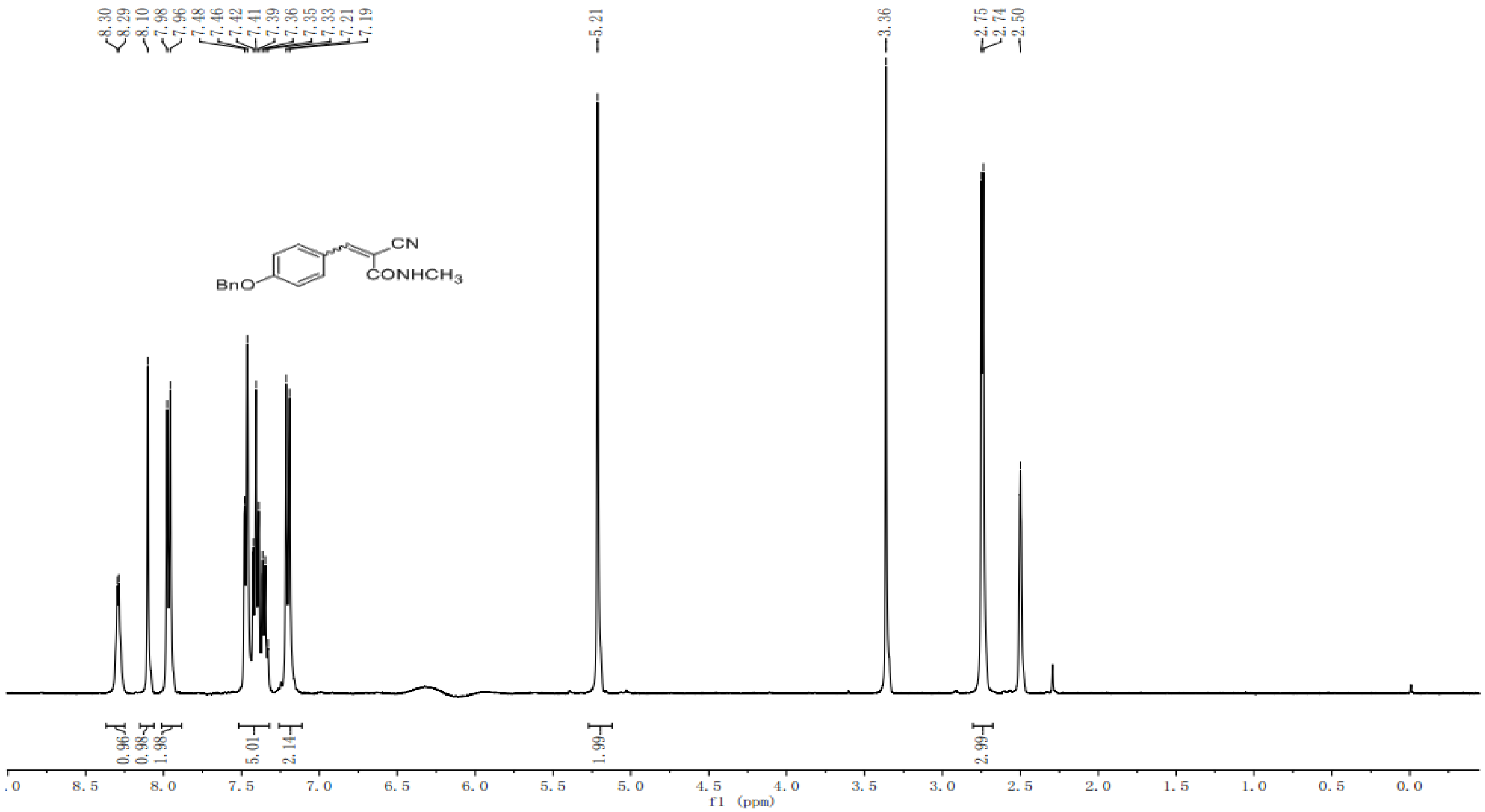

Figure S10. ^{13}C NMR spectrum of compound 13

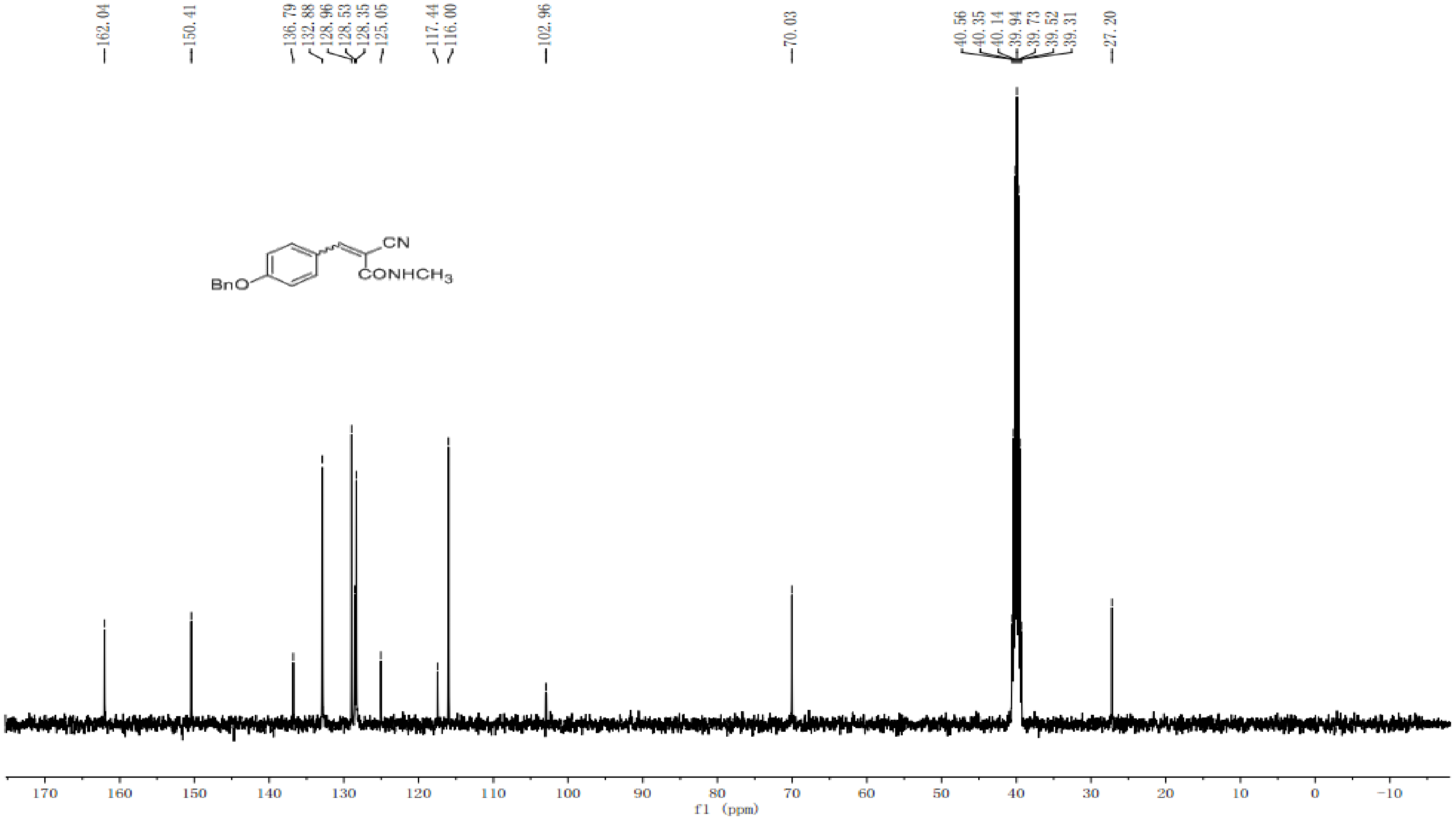

Figure S11. ^{1}H NMR spectrum of compound 14

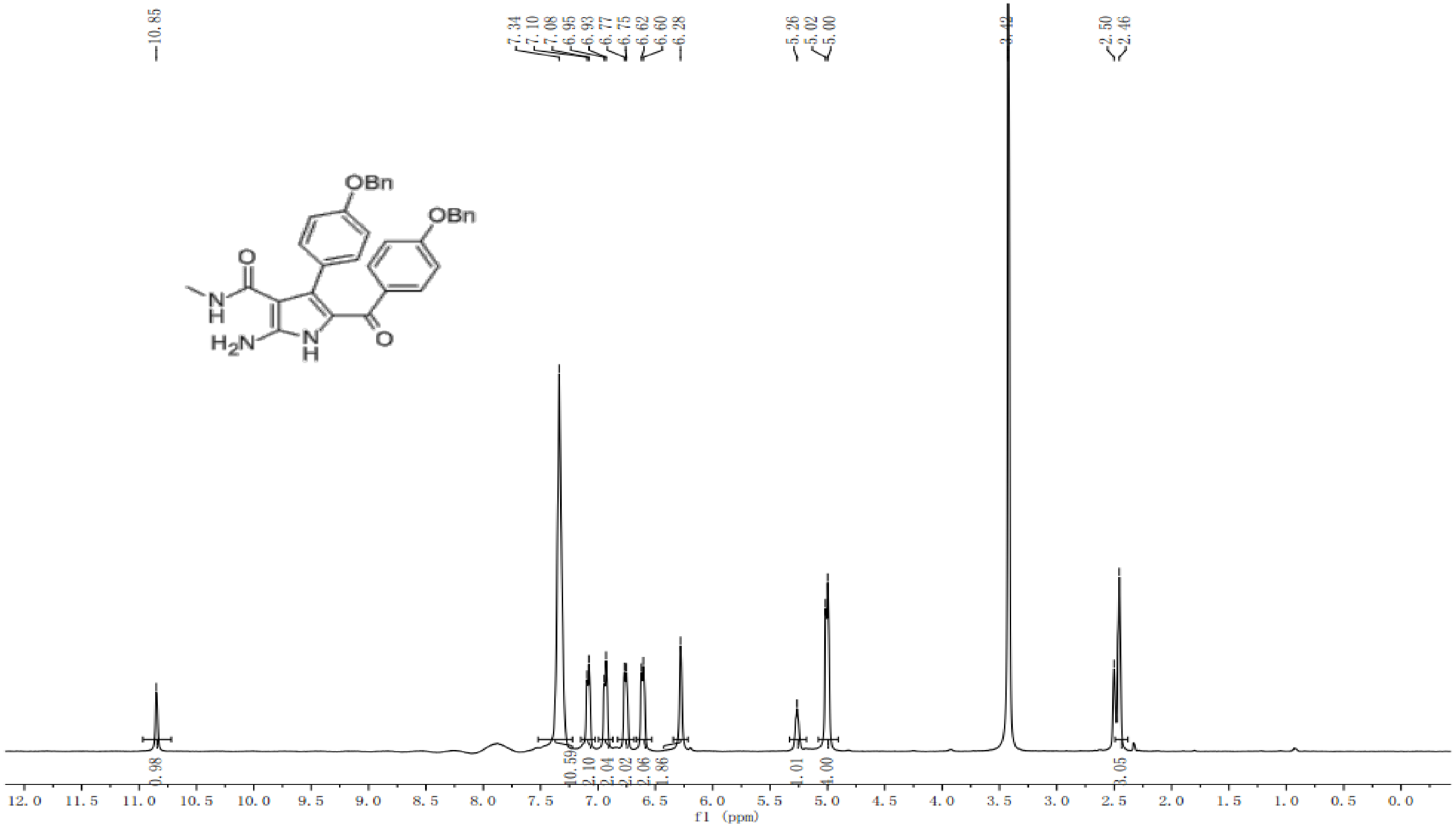

Figure S12. ^{13}C NMR spectrum of compound 14

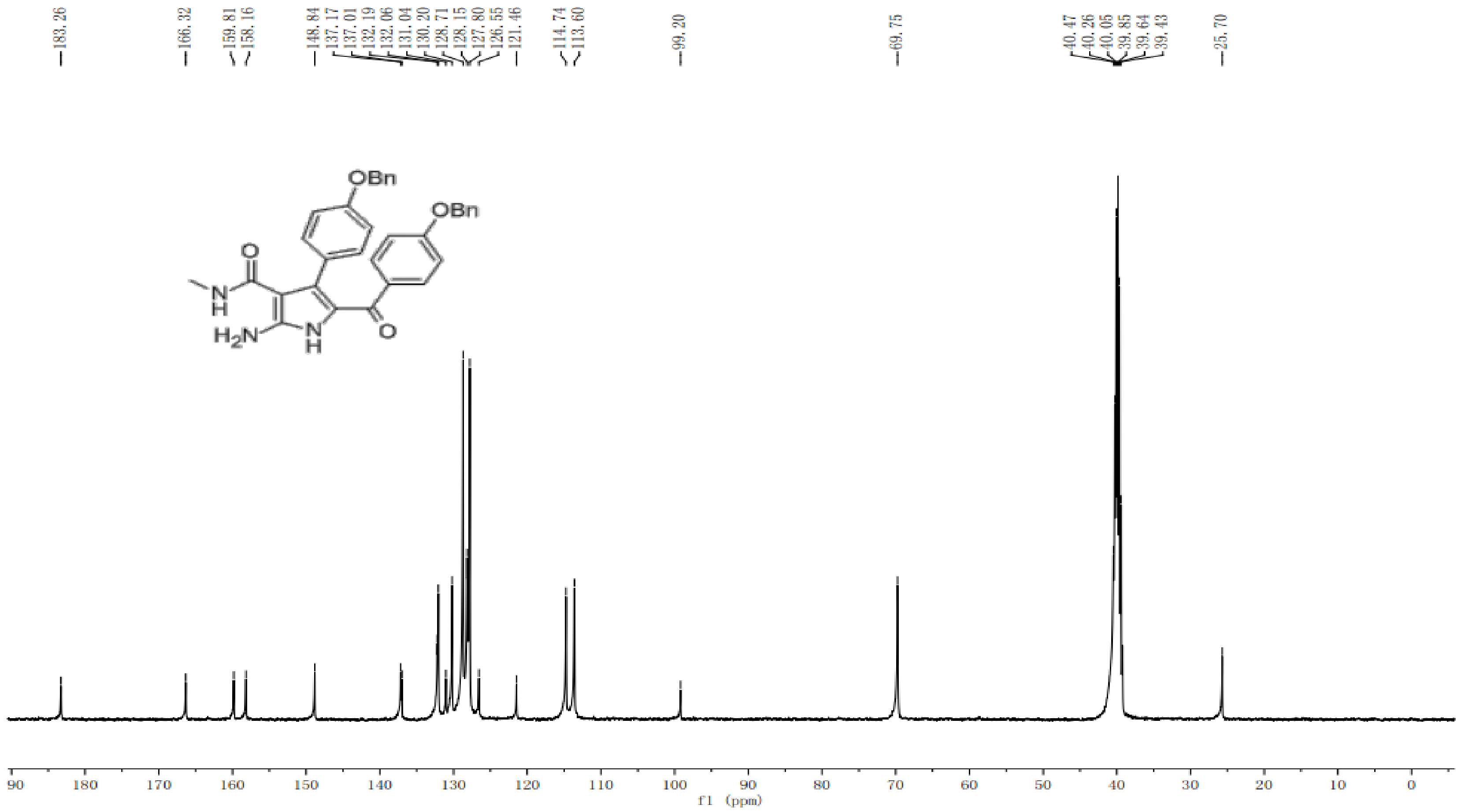

Figure S13. ^{1}H NMR spectrum of compound 15

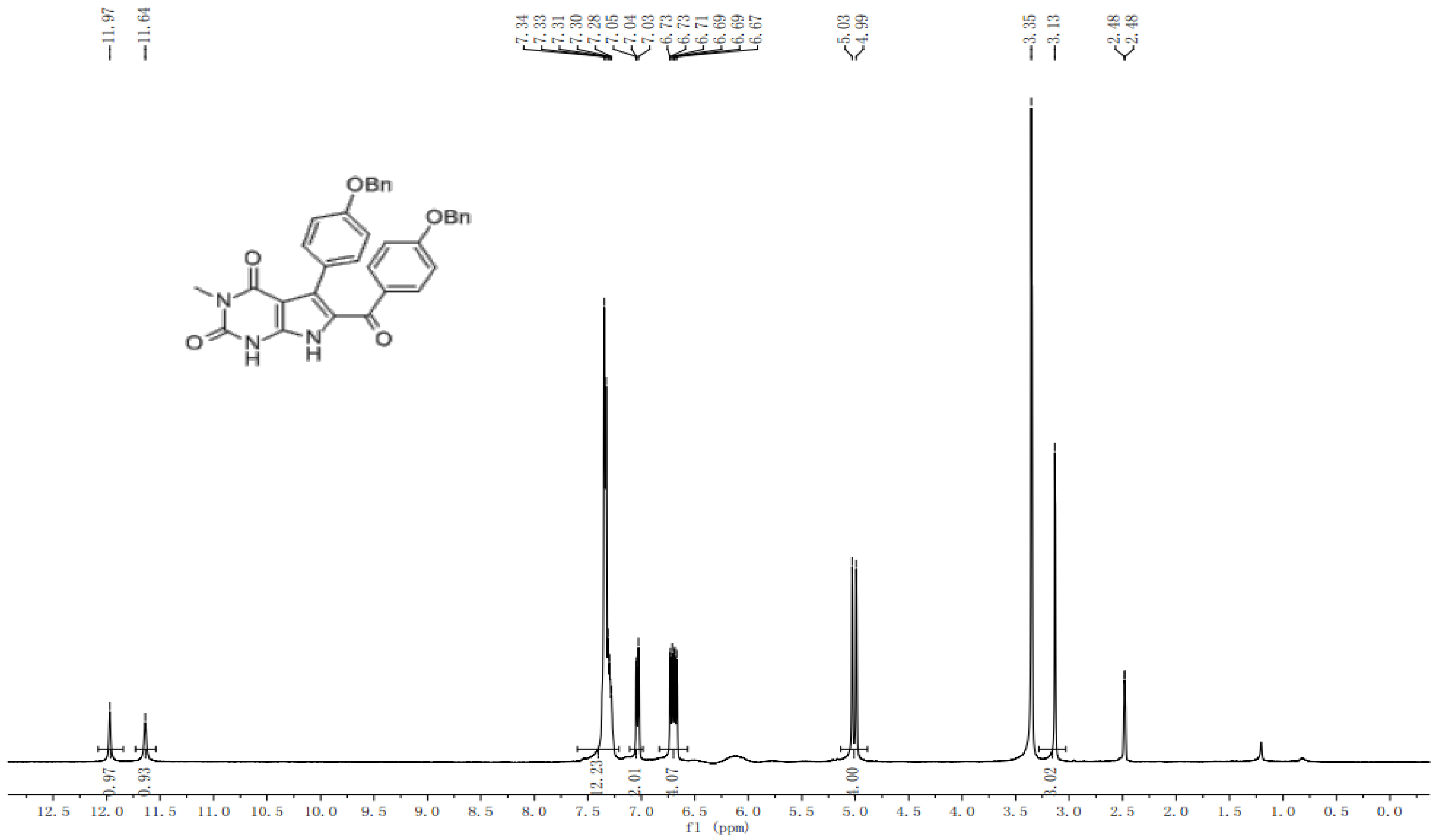

Figure S14. ^{13}C NMR spectrum of compound 15

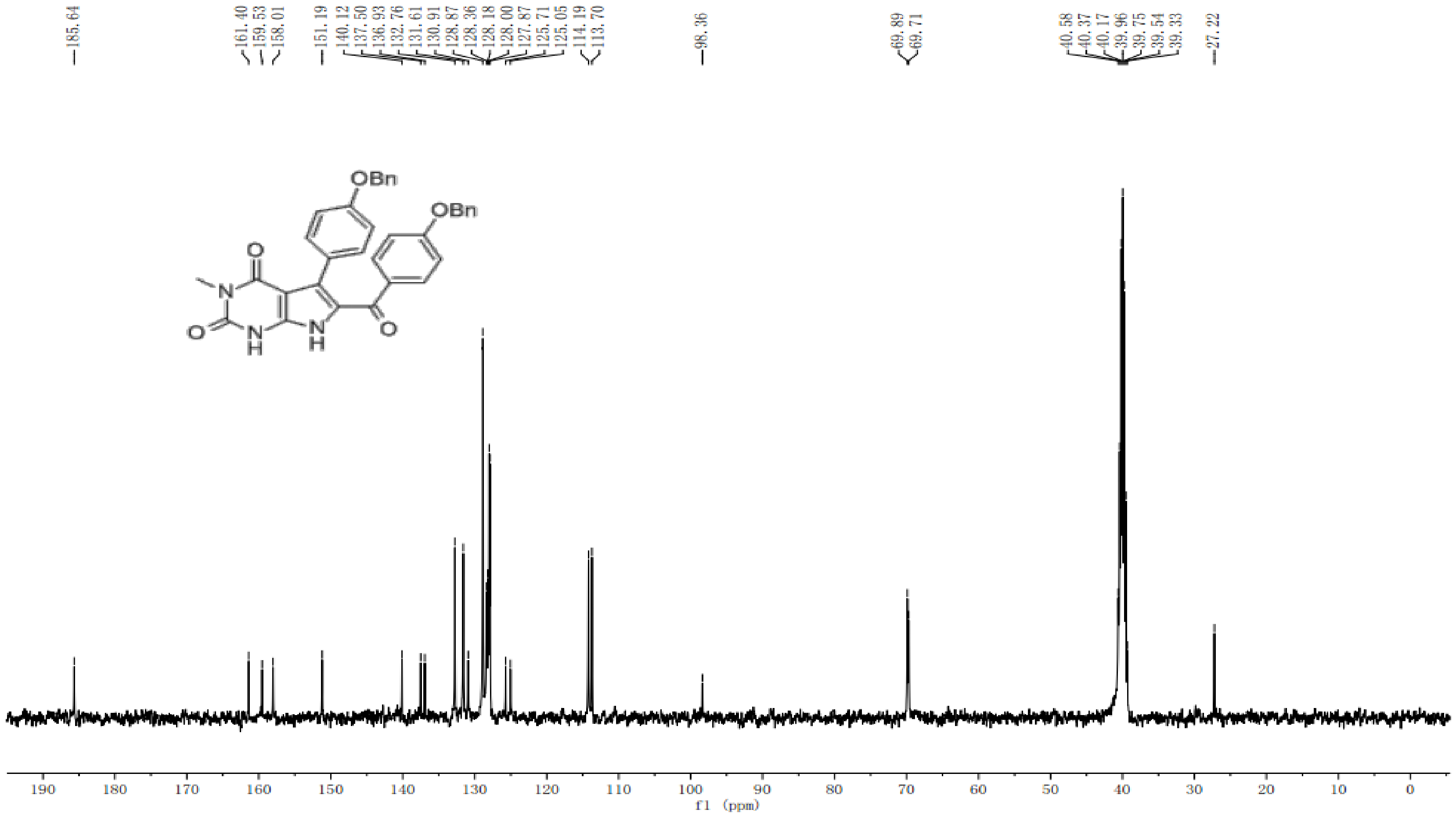

Figure S15. ^{1}H NMR spectrum of compound 5 (Rigidin E)

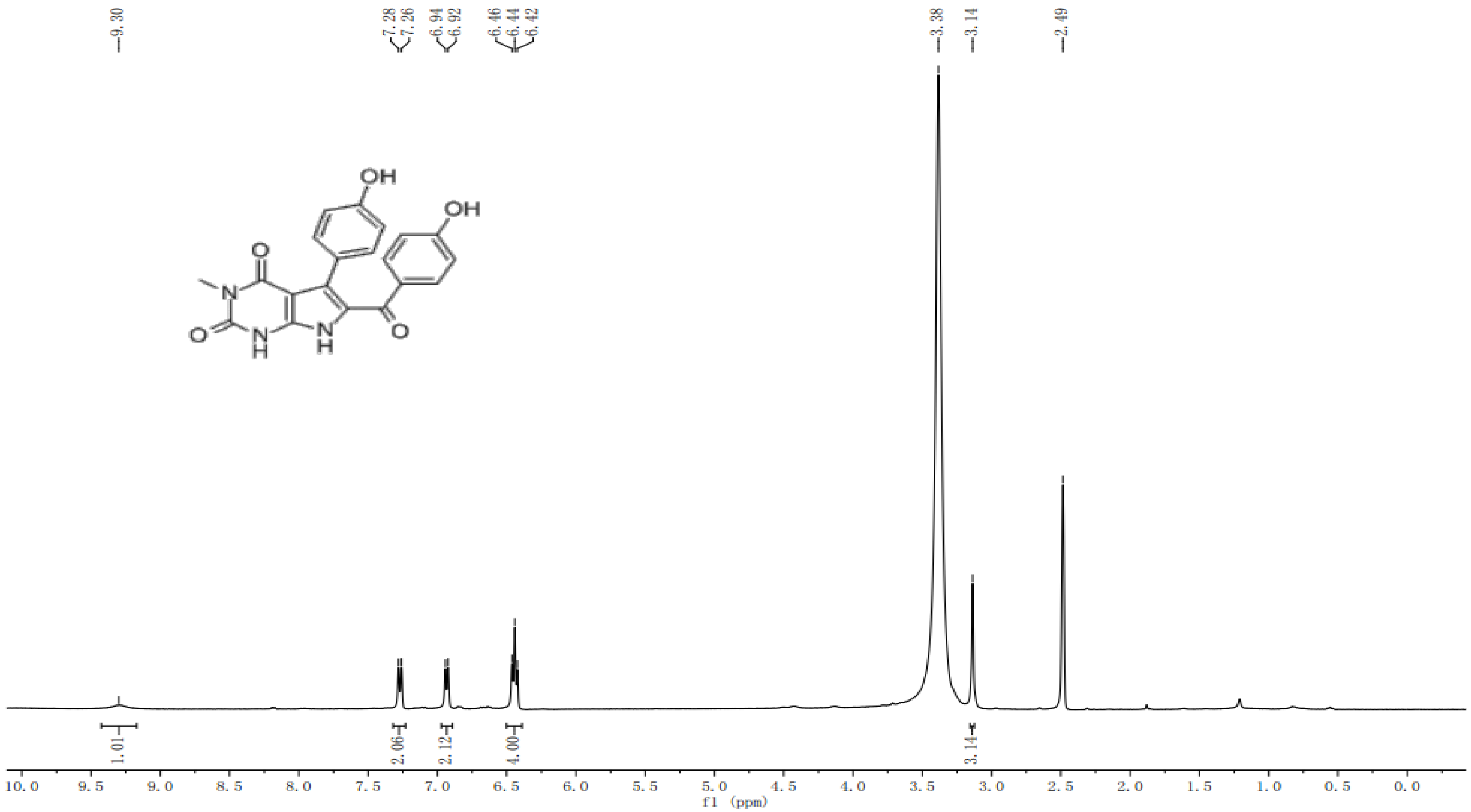

Figure S16. ^{13}C NMR spectrum of compound 5 (Rigidin E)

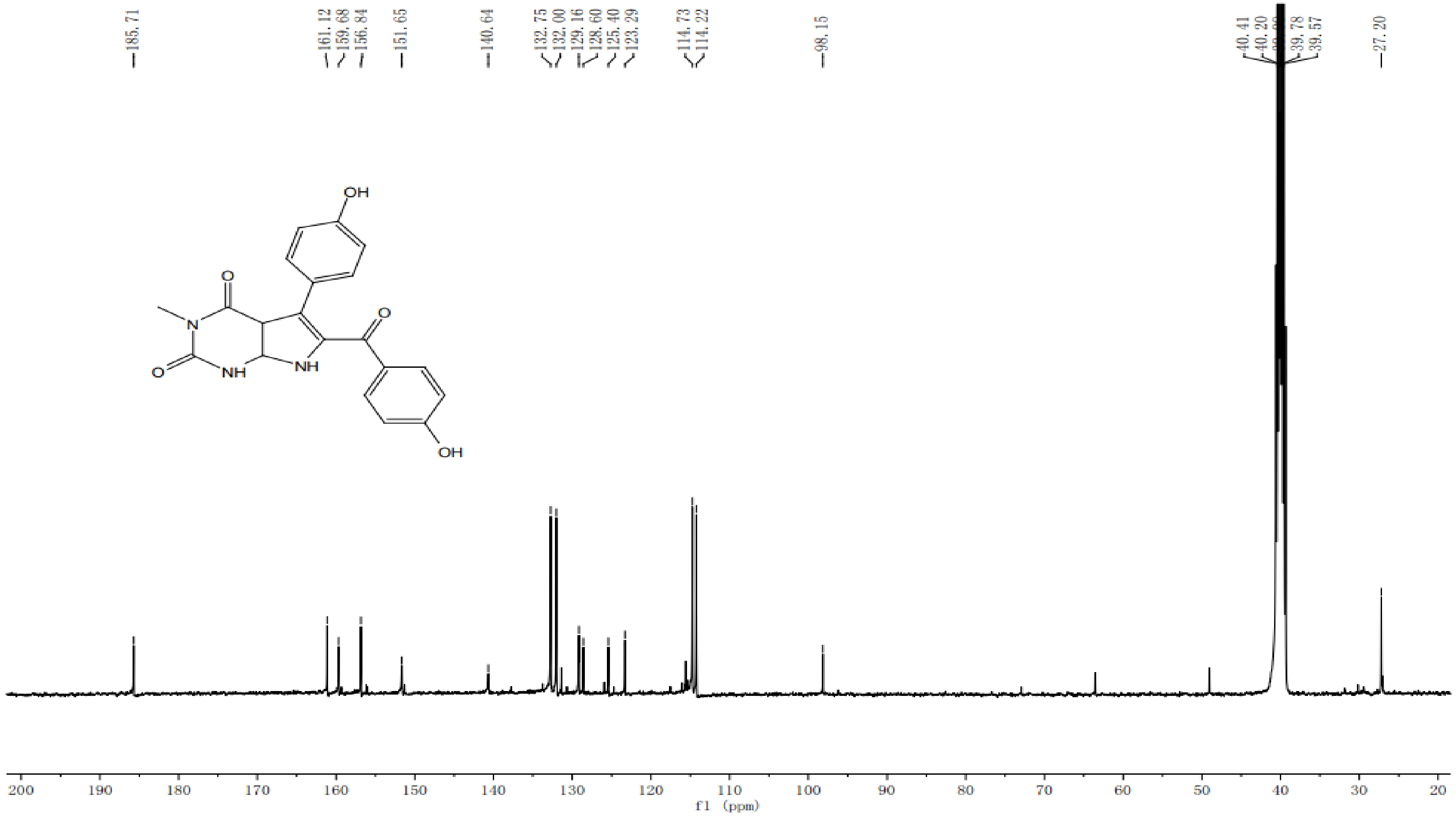

Supplement: Supplementary File 1: — PDF-Document (PDF, 689 KB) [file marinedrugs-10-01412-s001.pdf]
